# Podocyte Sphingolipid Signaling in Nephrotic Syndrome

**DOI:** 10.33594/000000356

**Published:** 2021-04-17

**Authors:** Guangbi Li, Jason Kidd, Todd W.B. Gehr, Pin-Lan Li

**Affiliations:** a Department of Pharmacology and Toxicology, School of Medicine, Virginia Commonwealth University, Richmond, VA, USA,; b Division of Nephrology, School of Medicine, Virginia Commonwealth University, Richmond, VA, USA

**Keywords:** Sphingolipid, Acid ceramidase, Sphingomyelin-like phosphodiesterase 3b, Podocyte, Nephrotic syndrome

## Abstract

Podocytes play a vital role in the pathogenesis of nephrotic syndrome (NS), which is clinically characterized by heavy proteinuria, hypoalbuminemia, hyperlipidemia, and peripheral edema. The pathogenesis of NS has evolved through several hypotheses ranging from immune dysregulation theory and increased glomerular permeability theory to the current concept of podocytopathy. Podocytopathy is characterized by dysfunction or depletion of podocytes, which may be caused by unknown permeability factor, genetic disorders, drugs, infections, systemic disorders, and hyperfiltration. Over the last two decades, numerous studies have been done to explore the molecular mechanisms of podocyte injuries or NS and to develop the novel therapeutic strategies targeting podocytopathy for treatment of NS. Recent studies have shown that normal sphingolipid metabolism is essential for structural and functional integrity of podocytes. As a basic component of the plasma membrane, sphingolipids not only support the assembly of signaling molecules and interaction of receptors and effectors, but also mediate various cellular activities, such as apoptosis, proliferation, stress responses, necrosis, inflammation, autophagy, senescence, and differentiation. This review briefly summarizes current evidence demonstrating the regulation of sphingolipid metabolism in podocytes and the canonical or noncanonical roles of podocyte sphingolipid signaling in the pathogenesis of NS and associated therapeutic strategies.

## Introduction

Sphingolipids, a class of lipids containing a backbone of sphingoid bases, are important components of the plasma membrane and thereby determine the structural and functional integrity of mammalian cells [1]. Sphingolipids contribute to the formation of a mechanically stable and chemically resistant outer leaflet of the plasma membrane lipid bilayer. The aggregation of sphingolipids, cholesterol, and proteins in plasma membrane leads to the construction of microdomains termed lipid rafts. These lipid rafts organize the assembly of signaling molecules and promote the interaction of protein receptors and their effectors, leading to the initiation and enhancement of the signal transduction [2]. Recently, it has been found that single sphingolipid metabolites, such as ceramide and sphingosine-1-phosphate (S1P), also mediate various cellular activities such as cell apoptosis, proliferation, stress responses, necrosis, inflammation, autophagy, senescence, and differentiation [3–8]. Sphingolipids have also been reported to be important regulators in podocytes.

As terminally differentiated epithelial cells, podocytes cover the outer surface of glomerular capillaries and form the glomerular filtration barrier in along with the glomerular basement membrane and glomerular endothelial cells. They typically do not proliferate. Many glomerular diseases in which the podocyte is the target of injury are not associated with podocyte proliferation [9, 10]. Therefore, the exploration of pathological mechanisms underlying podocyte dysfunction and depletion is essential for the development of therapeutic strategies which may prevent or slow down the progression of glomerular disease. The vital roles of sphingolipids and sphingolipid-modulating enzymes in the regulation of podocyte function has been well established in previous studies [11]. This review will focus on different types of sphingolipids and sphingolipid-modulating enzymes in podocytes that have been implicated in the pathogenesis of nephrotic syndrome (NS). We will also discuss the potential therapeutic strategies for NS related to sphingolipid signaling in podocytes.

## Sphingolipid Metabolic Pathways

Sphingolipids are a class of lipids which vary in hydrophobic and hydrophilic properties. The long chain sphingoid base, such as sphingosine, is the hydrophobic region of sphingolipids, which is linked to the acyl group of a fatty acid via an amide bond. The hydrophilic region of ceramide, a simple sphingolipid, is the hydroxyl group. The diversity of ceramides depends on the different lengths of the fatty acid acyl chain. As the center of sphingolipid metabolism, ceramide can be produced by sphingomyelinase-dependent hydrolysis of sphingomyelin. Another pathway for ceramide production is de novo synthesis, of which the condensation of palmitoyl-CoA and serine catalyzed by serine palmitoyl transferase is the initial step. Then, the reduction of 3-keto-dihydrosphingosine to dihydrosphingosine occurs, which is followed by acylation by ceramide synthase [12, 13]. Finally, a specific desaturase catalyzes the oxidation of dihydroceramide to ceramide. Another option for ceramide production is the breakdown of glycosphingolipid and galactosylceramide to dihydroceramide and subsequent hydrolyzation. Many essential biosynthetic pathways utilize ceramide as substrate. Also, numerous cellular responses are the consequence of ceramide accumulation, including cell growth arrest, apoptotic cell death, cell senescence, and stress response [14].

The transfer of phosphocholine from phosphatidylcholine to ceramide catalyzed by sphingomyelin synthase leads to the production of sphingomyelin as the most abundant sphingolipid in the plasma membrane [15]. In the generation of glycosphingolipids, ceramide as the backbone can be converted to glucosylceramide, the simplest glycosphingolipid, by glucosylceramide synthase [16]. The addition of a galactose moiety transforms glucosylceramide into lactosylceramide which can be converted to ganglioside, a vital component of membrane microdomain with a role in cell-cell recognition, adhesion, and signal transduction [17]. Furthermore, the phosphorylation of ceramide by ceramide kinase leads to the production of ceramide-1-phosphate (C1P), which can act as pro-inflammatory or anti-inflammatory sphingolipid under different conditions [18].

**Fig. 1.**
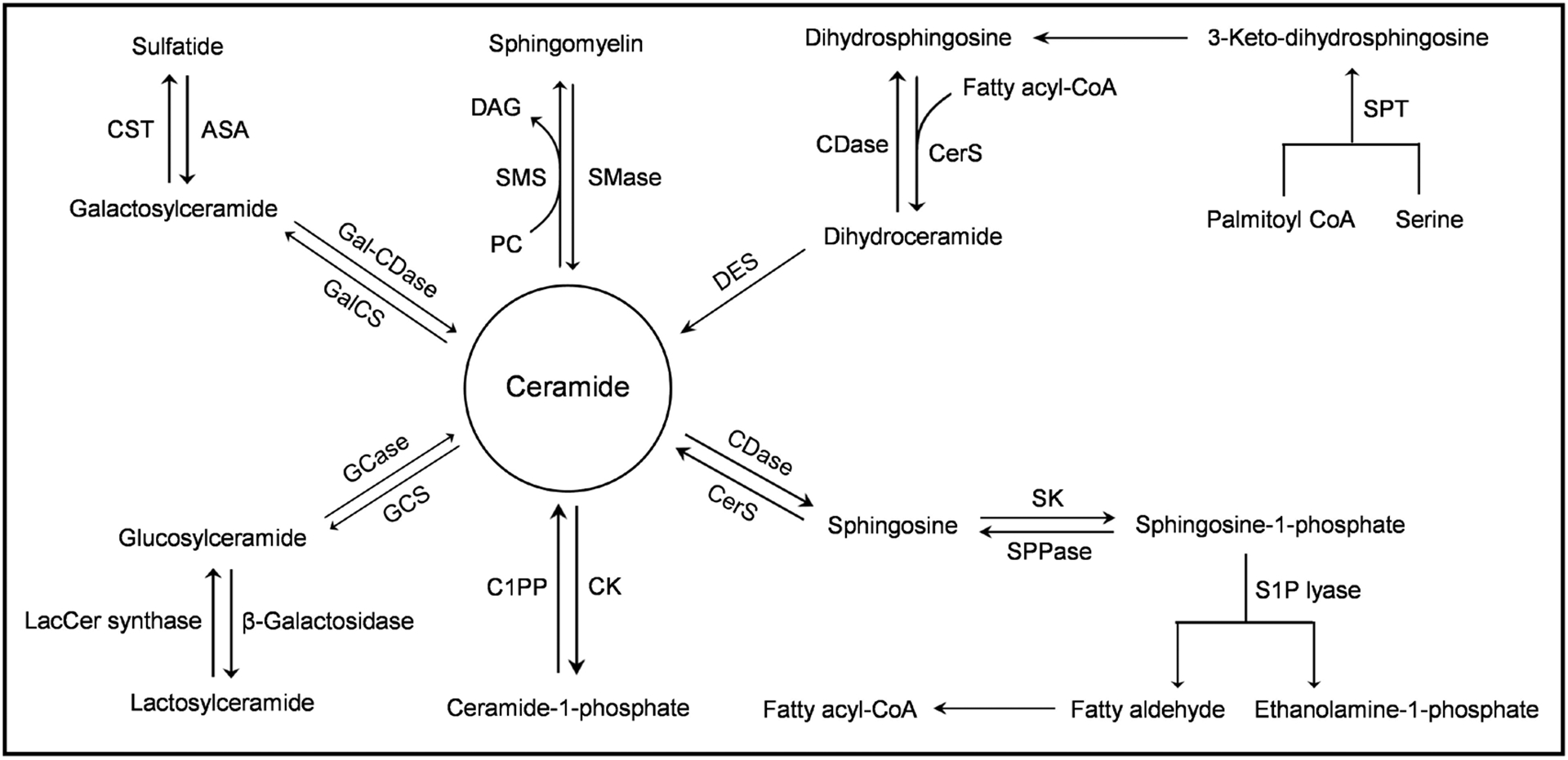
Sphingolipid metabolic pathways. De novo synthesis of ceramide consists of decarboxylation of a serine residue and condensation with a fatty acyl-CoA. Hydrolysis of SM by various SMases can also produce ceramide. The subsequent reactions catalyzed by CK, GCS, and GalCS lead to the production of other sphingolipids from ceramide. Many of these biochemical reactions are bidirectional. C1PP, ceramide-1-phosphate phosphatase; CDase, ceramidases; CK, ceramide kinase; CerS, ceramide synthase; DAG, diacylglycerol; DES, dihydroceramide desaturases; GalCS, galactosylceramide synthase; GCase, glucocylceramidase; GCS, glucosylceramide synthase; PC, phosphatidylcholine; S1P, sphingosine-1-phosphate; S1PP, S1P phosphatase; SK, sphingosine kinase; SMase, sphingomyelinase; SMS, sphingomyelinase synthase; SPL, S1P lyase; SPT, serine palmitoyl transferase.

Recent studies have shown that sphingolipid metabolites such as ceramides, sphingosine, and S1P serve as signaling molecules in many cellular activities including cell growth, differentiation, migration, and apoptosis [19, 20]. During the production of S1P, diacylation of ceramide to sphingosine by ceramidase and phosphorylation of sphingosine by sphingosine kinase (SK) occur in sequence [21–23]. Two isoforms of SK, namely SK1 and SK2, are ubiquitously expressed in mammalian cells. However, their intracellular locations and biological functions differ in certain types of cells [24, 25]. The irreversible breakdown of S1P to hexadecenal and phosphoethanolamine is catalyzed by S1P lyase [26]. Alternatively, the removal of phosphate moiety from S1P by S1P phosphatase leads to the generation of sphingosine, which can be either phosphorylated to S1P or utilized in sphingolipid salvage pathway for ceramide biosynthesis [27]. The role of S1P in the pathogenesis of podocyte injury and consequent glomerular disease remains controversial. Fig. 1 summarizes metabolic pathways of sphingolipids.

Sphingolipids can be generated and metabolized in the podocyte [1, 28–30]. These sphingolipids also importantly participate in the regulation of renal function and in the development of various kidney diseases. In recent studies, enhanced activities of acid sphingomyelinase (ASM) and increased ceramide production have been shown to play a pivotal role in mediating podocyte injury and glomerulosclerosis during hyperhomocysteinemia and obesity [31–33]. On the contrary, sphingosine has been reported to improve cell function in Niemann-Pick disease due to ASM gene mutations [34]. Mutations in the AC gene (ASAH1) or deficiency of lysosomal AC activity in human cells were found to be a major genetic or pathogenic mechanism for the development of Farber disease and partially for juvenile idiopathic arthritis that were shown to develop membranous nephropathy, focal segmental glomerulosclerosis (FSGS), and minimal change disease (MCD) [35–37]. More recently, mutations or deletion of sphingosine-1-phosphate lyase in humans and mice were reported to increase S1P and ceramide level in blood or tissues, which results in steroid-resistant NS with mesangial hypercellularity, glomerular hypertrophy and glomerular fibrosis [38–41]. We have also demonstrated that podocyte-specific Asah1 gene deletion induces podocytopathy and NS [42]. It seems that ceramide and associated sphingolipids may play a crucial role in the development of podocytopathy and NS. This led us to a major focus of this brief review on the physiological regulation of podocytes function and the pathophysiological role of sphingolipid in podocytopathy and NS.

## Classification of NS

A useful way to classify NS is based on the podocyte which is an important component of the glomerular filtration barrier. NS is defined by the presence of more than 3.5 grams of proteinuria daily with associated hypoalbuminemia, hyperlipidemia and peripheral edema. The pathogenesis of NS has evolved through several hypotheses ranging from immune dysregulation theory and increased glomerular permeability theory to the current concept of podocytopathy. Podocytopathy is a kidney disease in which direct or indirect podocyte injury drives proteinuria or NS. Podocytopathy may be caused by unknown permeability factor, genetic disorders, drugs, infections, systemic disorders, and hyperfiltration [43].

An unknown circulating factor can result in diffuse podocyte foot process effacement and proteinuria. Morphologically, FSGS and MCD are different descriptions of histologic lesions that are associated with diffuse podocyte foot process effacement. Ultimately, if not treated, patients with these diseases can develop end-stage renal disease (ESRD) and severe complications related to protein lost [44]. Podocytopathies due to unknown permeability factor can also be classified based on response to treatment with corticosteroids. A response to treatment is defined by the remission of proteinuria and improvement in renal function. Based on response to therapy, patients can be classified as steroid dependent or steroid resistant. Patients with steroid sensitive NS remit after several weeks of steroid usage though may ultimately be steroid dependent or require other immunosuppressant therapy. Patients with steroid resistant NS do not remit after several months of steroid use [45]. Until now, the mechanism by which corticosteroid therapy leads to remission of NS remains poorly understood. Also, the improvement of our understanding in the steroid resistance of some NS patients is much needed.

Although renal biopsy contributes to the classification of NS, there is no evidence that results of renal biopsy can provide accurate prediction of their response to corticosteroid therapy [46]. Histologic lesions can only reflect different patterns of podocyte injury instead of confirming the actual pathogenesis that may suggest the therapeutic strategy for patients. Different glomerular morphological patterns may be associated with the same genetic cause. On the contrary, the same patterns of glomerular morphological changes may be attributed to different genetic mutations [47, 48].

The treatment of patients with podocytopathies due to genetic mutations is limited to supportive management with blood pressure reduction including blockade of renin angiotensin system. For patients who develop podocytopathies due to toxic factors, removal of the offending agent is paramount. In some cases, these patients need short courses of immunosuppressive treatment. Patients with podocytopathies associated with systemic disorders require treatment of their underlying disorders to slow the progression of kidney disease [43, 49]. Pathogenesis-based classification of podocytopathies is summarized in Table 1.

## Pathophysiology of Podocytes in NS

Increasing evidence has indicated that podocyte plays a vital role in the pathogenesis of NS. The pathogenesis of podocytopathy may be attributed to a single genetic mutation or environmental risk factors due to systemic diseases. Alternatively, numerous genetic mutations and/or environmental risk factors may work together to initiate or enhance podocytopathy, leading to proteinuria or NS [50]. Previous studies have uncovered various environmental causes of NS in which podocyte dysfunction or injury plays a pivotal role [50]. In diabetes mellitus, glomerular hyperfiltration may lead to proteinuria and renal dysfunction. Enhanced proximal tubular reabsorption of glucose and sodium results in decreased afferent arteriolar resistance and increased single-nephron glomerular filtration rate (GFR) through the inhibition of tubuloglomerular feedback [51]. Consequently, elevation of GFR promotes podocyte stress and thereby induces foot process effacement and podocyte detachment, leading to early changes in glomerular function developing into diabetic nephropathy (DN) [51]. As another environmental cause, human immunodeficiency virus (HIV) infection may lead to podocytopathy associated with microcystic tubular dilatation [52]. In podocytes, interferon-mediated antiviral immune response to HIV infection enhances transcription of APOL1, the gene encoding apolipoprotein L1, leading to activation of inflammatory cell death [53]. Other infections have also been confirmed to directly induce podocyte injury, including amyloidosis, hepatitis C virus, parvovirus B19, and bisphosphonate [50]. In addition, drugs such as lithium and bisphosphonate have been implicated in the pathogenesis of podocytopathy [50].

To date, more than 50 genetic mutations have been identified which cause podocytopathies. The discovery of these genetic mutations as causes of podocytopathies, particularly steroid-resistant NS, has demonstrated the importance of corresponding proteins in the maintenance of podocyte integrity and glomerular function. For example, the identification of mutations in NPHS1, the gene encoding nephrin, and NPHS2, the gene encoding podocin, in some NS patients has confirmed the vital role of slit diaphragm proteins in structural and functional integrity of podocytes. The discovery of mutations in ACTN4, the gene encoding α-actinin-4, and ANLN, the gene encoding anillin, in certain NS patients has revealed that instability of actin cytoskeleton in podocytes may be a pathogenic mechanism of NS [54–58]. The involvement of these podocyte-associated proteins in the pathogenesis of podocytopathies confirms that targeting podocyte structural and functional integrity may be a novel therapeutic strategy for patients with NS, especially steroid-resistant NS. Below are several good examples:

### RhoA

The foot process effacement of podocytes is a canonical feature of NS. The shape and movement of podocyte foot process is regulated by the actin cytoskeleton. The dynamic control of actin cytoskeleton, such as polymerization and depolymerization, is mediated by more than a hundred proteins [59]. The essential tensile strength for a central core of filament bundles is provided by actin in a linear structure [60]. As one of the small GTPases of the Rho family (RhoA, Rac1, Cdc42) that is important in dynamic control of actin, Rac1 can activate Arp2/3 to enhance the formation of branched actin. Also, polymerization of actin at cell–cell junctions can be induced by Rac1 [61]. The cycle of Rho GTPases is that they are active when bound to GTP and inactive when bound to GDP [62]. The dynamic switch between two distinct conformational states of these small GTPases allows them to modulate podocyte actin cytoskeleton and cell-cell adhesion with spatial and temporal precision. In a mouse model with podocyte-specific transgene of RhoA in a doxycycline-inducible constitutively active form, enhanced actin cytoskeleton polymerization, decreased nephrin expression, and apoptosis were observed in podocytes [63]. On the contrary, the reduction of podocyte stress fiber was found in mice with podocyte-specific transgene of RhoA in a dominant negative form [63]. In another study, albuminuria, podocyte foot process effacement, and FSGS were attributed to the enhancement of RhoA activity [64]. The inhibition of RhoA expression, however, led to podocyte foot process effacement in mice while no morphological changes of glomeruli were observed under light microscope [64]. These findings indicate that the function of RhoA in a normal range is essential for maintaining the structural integrity of podocytes. Abnormality of RhoA activity may lead to podocyte injury and NS.

### Actinin

As an actin-bundling protein, α-actinin-4 is vital for maintaining actin cytoskeleton integrity and adhesion property of podocytes. It has been reported that mutations in ACTN4, the gene encoding α-actinin-4, caused adult-onset autosomal dominant FSGS [65]. The inhibition of actin assembly at the junctional complexes is attributed to a point mutation of α-actinin-4 (K255E) during FSGS [66]. This point mutation can also enhance the affinity of α-actinin-4 to actin, leading to aggregation of α-actinin-4 and misfolded actin in podocytes [67]. Another point mutation of α-actinin-4 (K256E) has been demonstrated to result in increased targeting of mutant protein for degradation, impairment of the ubiquitin–proteasome system, increased endoplasmic reticulum stress, and exacerbation of apoptosis in podocytes [68].

### Nephrin

Nephrin is a slit diaphragm protein encoded by NPHS1, which is essential for the normal function of podocytes [69]. In 1998, mutations in NPHS1 were found to induce congenital NS of the Finnish type, which is characterized by severe proteinuria in utero. As a transmembrane protein, nephrin is composed of eight extracellular immunoglobulin domains, a fibronectin III domain, and an intracellular domain with several tyrosine residues [70]. Fyn-dependent phosphorylation of tyrosine residues of nephrin is followed by the binding of Nck adapter proteins, which maintains the structural integrity of actin cytoskeleton in podocytes via interaction with N-WASP and p21-activated kinases (PAKs) [71, 72]. Also, it has been demonstrated that actin cytoskeleton in podocytes can be determined by the interaction between nephrin and IQ motif containing GTPase activating protein, an effector protein for Rac1 and Cdc42 [73]. In 2000, podocin, another slit diaphragm protein was discovered [74]. Mutations in NPHS2, the gene encoding podocin, are a frequent cause of NS in families with congenital and infantile NS [75]. The lipid recognition motif localizes podocin to lipid rafts in the slit diaphragm of podocytes. Also, in these lipid microdomains, nephrin can be recruited and stabilized by podocin, which is associated with the enhancement of its function [76].

### CD2-associated protein

CD2-associated protein (CD2AP) as a membrane protein is critical for stabilizing the interaction between T-cells and antigen-presenting cells. This protein has been linked to the regulation of podocyte slit diaphragm [77]. In a study of CD2AP-deficient mice, death occurred due to renal failure. In wild type mice, CD2AP was mainly expressed in podocytes. The association of CD2AP and nephrin may be essential for the preservation of podocyte slit diaphragm. CD2AP deficient mice developed podocyte foot process effacement, leading to mesangial cell hyperplasia and extracellular matrix deposition [77]. Another study has reported that CD2AP can be recruited to lipid rafts with podocin, which further confirms its role in the regulation of podocyte slit diaphragm [78]. More recently, it has been found that the upregulation of cytosolic cathepsin L due to lack of CD2AP may result in the proteolysis of synaptopodin, dynamin, and RhoA in podocytes, leading to actin cytoskeleton remodeling and hypersensitivity to transforming growth factor-β-induced apoptosis [79].

### TRPC6

Transient receptor potential 6 (TRPC6) channel is a Ca^2+^-permeable nonselective cation channel which interacts with nephrin and podocin at the slit diaphragm. The late-onset autosomal dominant FSGS has been found to be induced by pathological elevation of Ca^2+^ influx due to mutations in TRPC6 [80]. It has been reported that TRPC6 channel-dependent membrane stretch detection may cause remodeling of actin cytoskeleton to a contractile state [81]. Although regular Ca^2+^ influx through the TRPC6 channel is important for maintaining normal function of RhoA and inhibiting podocyte migration, the excess of TRPC6 channel-mediated Ca^2+^ influx may attenuate the flexibility of podocytes in response to environmental changes, leading to disorganization of stress fiber and actin cytoskeleton [82].

### Palladin

As a cytoskeletal protein with essential functions for stress fiber formation, palladin has been detected in various tissues, including the kidney [83, 84]. In previous studies, it has been found that palladin interacts with α-actinin through a novel α-actinin binding motif in the N-terminal half of palladin [85]. Moreover, palladin has been confirmed to colocalize with vasodilator-stimulated phosphoprotein (VASP) and α-actinin-1 in dense regions along stress fibers and in focal adhesions [83]. Palladin also plays an important role in actin dynamics of podocytes [86]. In murine podocytes, the colocalization of palladin and F-actin has been observed in dense regions of stress fibers and motile cell margins and during focal adhesions and cell–cell contacts. Interestingly, inhibition of palladin expression was found to decrease the formation of ring-like structure of F-actin in podocytes [86]. Moreover, podocytes transfected with palladin siRNA had decreased actin filament staining, smaller focal adhesions, and reduction of the podocyte-specific proteins synaptopodin and α-actinin-4 [87]. In podocyte-specific palladin knockout mice, abnormal glomerular morphology and reduction of nephrin and vinculin in podocytes were observed [87]. Clinically, kidney biopsy specimens from patients with DN and FSGS have shown the reduction of palladin expression in podocytes [87]. Taken together, these findings indicate that palladin plays an important role in the regulation of the actin cytoskeleton and slit diaphragm of podocytes. Genetic mutations or downregulation of palladin may also be implicated in the pathogenesis of NS. Further studies are needed to confirm whether palladin can be a therapeutic target against NS.

## Podocyte Sphingolipids and Metabolizing Enzymes in NS

The accumulation of various sphingolipids in podocytes has been found in several models of experimental and clinical NS. Also, it has been reported that development of NS in several models may be attributed to sphingolipid accumulation in podocytes in the absence of genetic mutations. Here, we highlight several sphingolipids and modulating enzymes which may be essential for the maintenance of podocyte homeostasis or implicated in the onset or development of NS.

### Ganglioside

In 1978, it has been found that proteinuria is associated with glycosphingolipiduria during various etiologies [88]. A specific glycosphingolipid, ganglioside GM3 (GM3), functions as a receptor for soluble vascular endothelial growth factor receptor 1 (Flt1), which has been found to locate at lipid microdomains in the slit diaphragm of podocytes [89]. The conservation of podocyte actin cytoskeleton by Flt1 depends on its binding to GM3, which is essential for the prevention of proteinuria [89]. Also, podocyte-specific deletion of Flt1 has been demonstrated to induce NS in mice [89]. Enzymes catalyzing the sialylation of GM3, uridine diphospho-N-acetylglucosamine 2-epimerase and N-acetylmannosamine kinase, have been confirmed to be vital for maintenance of glomerular function and prevention of proteinuria, indicating the importance of GM3 [90]. GM3 plays an important role in regulation of podocyte actin cytoskeleton and slit diaphragm.

Other gangliosides may also affect podocyte function and be involved in the pathogenesis of NS. For example, O-acetylated disialosyl lactosylceramide (GD3) has been confirmed to be a podocyte-specific ganglioside [91]. Phosphorylation of nephrin and consequent translocation of nephrin from slit diaphragm to cytosol were observed in podocytes treated with the antibody against O-acetylated GD3 [92]. Moreover, reduction of O-acetylated GD3 was associated with NS in rats after injection of puromycin [93]. Based on these results, the spatial specificity of GD3 in podocytes renders it a therapeutic target for treatment of NS. The potential role of other gangliosides in the regulation of podocyte function or in the development of podocytopathy will be an interesting avenue for further research.

### Globotriaosylceramide

Fabry disease, a lysosomal storage disease, is caused by mutations in the gene encoding α-galactosidase A (α-GLA) leading to the systemic accumulation of globotriaosylceramide (Gb3) and related glycosphingolipids in the brain, heart, and kidney [94]. In the plasma or urine of patient’s with Fabry disease, increased levels of Gb3 and globotriaosylsphingosine were detected [95–98]. In renal cells, the accumulation of Gb3 mainly occurred in lysosomes, endoplasmic reticulum, and nuclei [99]. During Fabry disease, podocytes may develop hypertrophy, foamy appearing vacuoles, and characteristic inclusion bodies of glycolipids, which are associated with mesangial widening in glomeruli [100]. There is clinical evidence showing that podocyte foot process effacement is attributed to the elevated Gb3 level in podocytes during Fabry disease [101, 102]. The most effective treatment for Fabry disease is enzyme replacement therapy using recombinant human α-GLA, which has been shown to inhibit the development of NS and prevent renal failure in these patients [101, 103]. In α-GLA knockout mice, the accumulation of Gb3 and reduction of glucosylceramide were observed in plasma, liver, spleen, heart, and kidney. Also, podocytopathy was diagnosed in these mice. The level of glucosylceramide normalized by enzyme replacement therapy via recovered metabolism of Gb3 by α-GLA, however, reversed podocyte injury in these mice, confirming that α-GLA-dependent metabolism of Gb3 is essential for the preservation of podocyte integrity and prevention of NS [103]. In addition, it has been reported that the endocytosis of α-GLA into podocytes is mediated by endocytic receptors, megalin, sortilin, and mannose-6-phosphate receptor, which is the molecular basis of enzyme replacement therapy for NS associated with Fabry disease [104]. More recently, inhibition of autophagy has also been found to mediate the pathogenesis of podocyte injury due to knockdown of α-GLA. The evidence showed that intracellular accumulation of Gb3 due to knockdown of α-GLA attenuated the activity of mTOR kinase, leading to dysregulation of autophagy in podocytes [105]. Since the regular autophagy in podocytes has been demonstrated to be important for prevention of foot process effacement, proteinuria, and NS [106], dysregulation of autophagy may be another important mechanism leading to podocytopathy. Indeed, there is evidence that autophagic deficiency induces podocyte dedifferentiation [107].

### Acid Ceramidase

Farber disease is a genetic disorder caused by mutations in the gene encoding acid ceramidase (AC) on human chromosome 8p22. This enzyme catalyzes the hydrolysis of ceramide into sphingosine and free fatty acids. In patients with Farber disease, the accumulation of ceramide and associated sphingolipids was observed in many tissues including kidney. Ceramide accumulation in the kidney causes a particular phenotype of lipogranulomatosis [108]. For a long time, it remains unknown whether AC-dependent ceramide metabolism is essential for the conservation of podocyte function and whether the functional deficiency of AC induces podocyte injury and NS. Recently, the progress of understanding the physiological and pathophysiological roles of AC in podocytes have been made in several studies including those done in our laboratories.

One example is the important role of AC in DN. To our knowledge, DN is the most common cause of ESRD worldwide [109]. The disarrangement of lysosomal function is implicated in the initiation of podocyte injury and the development of DN [110]. A recent study has demonstrated that rapamycin as a lysosome function enhancer effectively attenuated STZ-induced DN via inhibition of podocyte apoptosis [111]. Also, the accumulation of ceramide and sphingomyelin due to STZ-induced de novo synthesis were attenuated by rapamycin. These findings indicate that the therapeutic effect of rapamycin on DN may be attributed to the suppression of abnormal sphingolipid metabolism [111]. More recently, the activation of adiponectin receptors has been found to regulate the expression of lysosomal AC that converts ceramide to sphingosine [112]. Both adiponectin receptor and lysosomal AC significantly decreased in podocytes of diabetic mice, which were attenuated by AdipoRon, an adiponectin receptor agonist. In addition, AdipoRon enhanced the activity of lysosomal AC and thereby inhibited ceramide accumulation in podocytes, which may contribute to the therapeutic effects of AdipoRon on DN [112]. Correspondingly, a clinical study has shown that the development of DN is associated with the elevation of urinary ceramide, which may be attributed to the abnormal sphingolipid metabolism by lysosomal enzymes in glomerular cells, such as podocytes [113]. It is clear that lysosomal sphingolipid metabolism is crucial for the maintenance of podocyte function and prevention of NS.

**Fig. 2.**
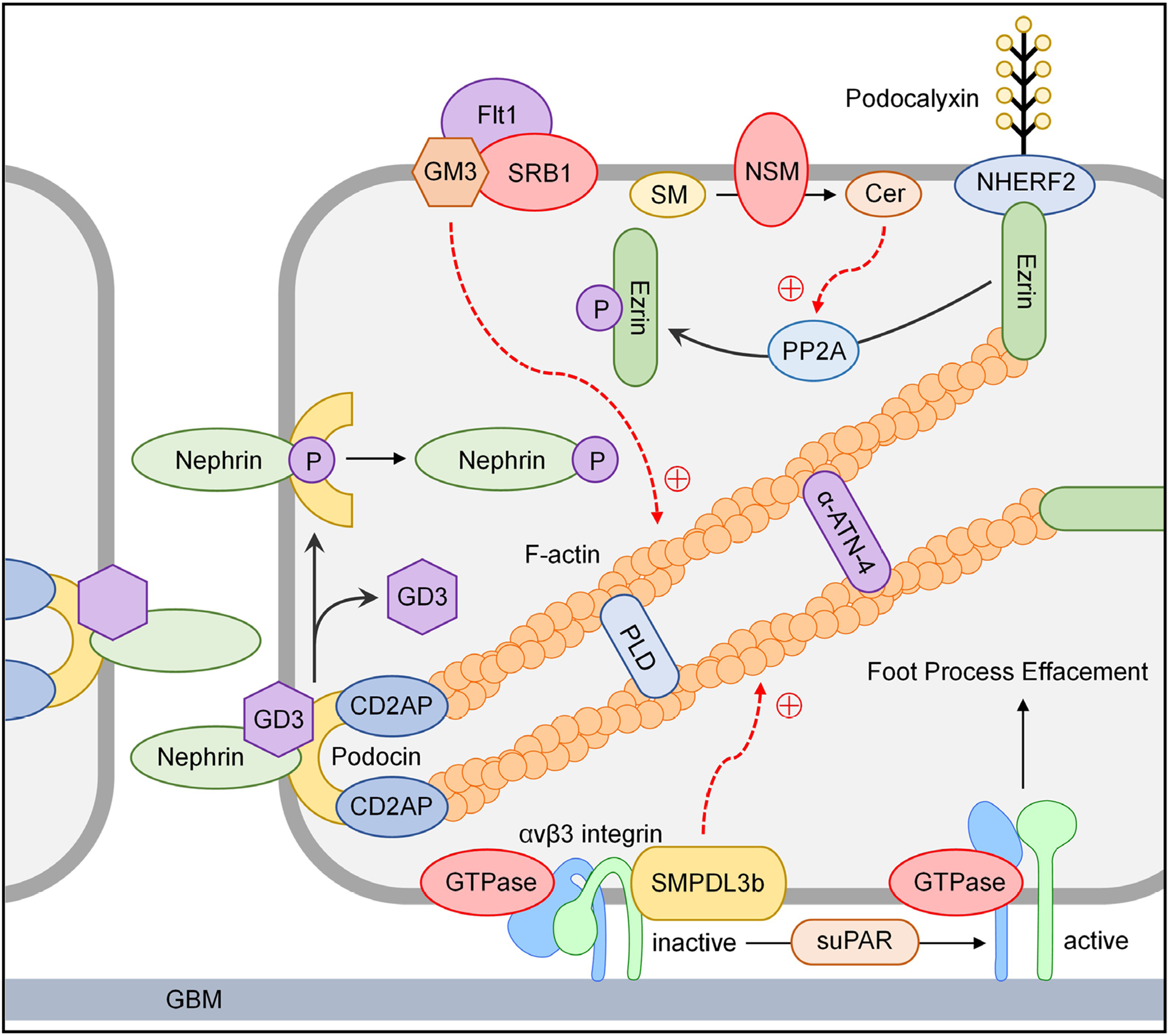
Regulation of slit diaphragm and actin cytoskeleton by sphingolipids and modulating enzymes in podocytes. Normal level of GD3 is essential for the maintenance of slit diaphragm in podocytes. Lack of GD3 leads to enhanced phosphorylation of nephrin, leading to increased translocation of nephrin to cytosol. GM3, together with Flt1 and SRB1, plays an important role in the regulation of actin cytoskeleton in podocytes. Overproduction of ceramide by NSM enhances the phosphorylation of Ezrin by PP2A, leading to actin cytoskeleton remodeling in podocytes. The expression of SMPDL3b on plasma membrane is vital for the maintenance of actin cytoskeleton in podocytes. Elevated suPAR is associated with reduction of SMPDL3b on plasma membrane, leading to actin cytoskeleton remodeling in podocytes. GD3, O-acetylated disialosyllactosylceramide; GM3, ganglioside GM3; Flt1, vascular endothelial growth factor receptor 1; SRB1, scavenger receptor class B type 1; NSM, neutral sphingomyelinase; PP2A, protein phosphatase 2A; suPAR, soluble urokinase plasminogen activator receptor.

Podocytes are highly differentiated cells which normally do not proliferate. Therefore, the normal function of lysosome and associated autophagic flux importantly preserve structural and functional integrity of podocytes during their long-term survival [114–117]. Lysosomal function has also been implicated in the regulation of multivesicular body (MVB) fate that determines the excretion of exosomes, one of extracellular vesicles (EVs) [110]. EVs or exosomes have been extensively studied for their biogenesis and related function in cell-to-cell communication and in the pathogenesis of different diseases including renal diseases [118–120]. In the kidneys, exosomes are not only a biomarker indicating kidney function or disease, but also serve as a mediator of intra-renal cell-to-cell communication, which may contribute to the development of various kidney diseases [119]. There is evidence that exosomes containing podocalyxin/podoplanin (podocyte-derived) increased in diabetic mice even before onset of albuminuria [121]. In some patients with NS, podocyte-derived exosomes increased along with albuminuria and glomerular degeneration [119, 122–126]. Recently, we have demonstrated that lysosomal AC controls lysosome function and exosome release in podocytes via regulation of transient receptor potential mucolipin 1 (TRPML1) channel-mediated Ca^2+^ release [127]. The precursor of ceramide, sphingomyelin was found to inhibit lysosomal Ca^2+^ release through the TRPML1 channel. On the contrary, the product of ceramide metabolism by AC, sphingosine enhanced TRPML1 channel-mediated Ca^2+^ release. This led us to hypothesize that lysosomal AC dysfunction may lead to podocyte injury. In a recent study, we developed a podocyte-specific AC gene knockout (Asah1^fl/fl^/Podo^cre^) mouse strain to test this hypothesis. It was found that podocyte-specific Asah1 gene deletion caused severe proteinuria and albuminuria in Asah1^fl/fl^/Podo^cre^ mice. Surprisingly, no significant morphological changes in glomeruli were observed in these mice under light microscope. Transmission electron microscopic analysis showed distinctive foot process effacement and microvillus formation in podocytes of Asah1^fl/fl^/Podo^cre^ mice. These functional and morphologic changes indicate the development of NS in these mice [42]. Ceramide accumulation determined by liquid chromatography-tandem mass spectrometry (LC/MS) was confirmed in isolated glomeruli of Asah1^fl/fl^/Podo^cre^ mice compared with their littermates. By crossbreeding Asah1^fl/fl^/Podo^cre^ mice with Smpd1^−/−^ mice, we produced a double knockout strain, Smpd1^−/−^/Asah1^fl/fl^/Podo^cre^, that also lacks Smpd1, the gene encoding ASM, to test whether reduction of ceramide production by ASM can reverse podocytopathy or NS induced by AC deficiency. These mice exhibited significantly lower level of glomerular ceramide and attenuated podocyte injury compared with Asah1^fl/fl^/Podo^cre^ mice [42]. Interestingly, we also demonstrated the elevation of exosome release from podocytes in Asah1^fl/fl^/Podo^cre^ mice [128]. Exogenous administration of sphingosine was shown to attenuate urinary exosome excretion via enhancement of TRPML1 channel-mediated Ca^2+^ release in podocytes. In this regard, previous studies have shown that elevation of podocyte-derived exosome release is associated with albuminuria and glomerular degeneration in NS patients [119, 124]. Based on these findings, it is possible that podocyte-derived exosomes due to AC deficiency may initiate or enhance podocyte injury in Asah1^fl/fl^/Podo^cre^ mice due to the dysregulation of TRPML1 channel activity. Fig. 2 summarizes the regulation of lysosome function by sphingolipids and associated metabolizing enzymes in podocytes and their implications in the maintenance of podocyte function and pathogenesis of podocytopathy and NS.

### Sphingomyelin-Like Phosphodiesterase 3b

Focal segmental glomerulosclerosis (FSGS) is a leading cause of proteinuria. Idiopathic FSGS may reoccur after kidney transplantation in approximately one-third of patients [129–131]. Recently, sphingomyelin-like phosphodiesterase 3b (SMPDL3b), an enzyme with structural homology to ASM, has been reported to play an important role in the pathogenesis of FSGS after kidney transplantation. A study on 41 patients after kidney transplantation showed that the ratio of SMPDL3b-positive podocytes in post-reperfusion biopsies remarkably decreased in patients who developed recurrent FSGS [132]. Treatment of human podocytes with the sera from patients with recurrent FSGS led to reduction of SMPDL3b expression and ASM activity in these cells, which were associated with actin cytoskeleton remodeling and apoptosis [132]. On the contrary, these pathological changes in podocytes were prevented by the overexpression of SMPDL3b or treatment with rituximab, a monoclonal antibody against CD20. The molecular mechanism by which rituximab protects podocytes from injury induced by sera from patients with recurrent FSGS may involve the stabilization of SMPDL3b in these cells [132]. The ratio of podocytes characterized by actin cytoskeleton remodeling due to the loss of stress fibers is in correlation with proteinuria, indicating a vital role of actin cytoskeleton remodeling in the pathogenesis of FSGS after kidney transplantation [132]. Correspondingly, another study has shown that rituximab prevents the disruption of pig podocytes and the early development of proteinuria after xenogeneic kidney transplantation in baboons in a SMPDL3b-dependent manner [133].

Interestingly, SMPDL3b expression level has been shown to determine the type of podocyte injury under different pathological conditions [134]. In FSGS, increased circulating soluble urokinase plasminogen activator receptor (suPAR) together with low or absent SMPDL3b expression were demonstrated to result in αVβ3 integrins activation, increased Src phosphorylation, and enhancement of Rac1 activity, which ultimately induced a migratory podocyte phenotype. In DN, however, elevated circulating suPAR was associated with the presence of high SMPDL3b expression, leading to competitive binding of SMPDL3b to suPAR, RhoA activation, and apoptosis in podocytes [134]. In db/db mice with podocyte-specific SMPDL3b gene deletion, podocyte injury was prevented, further confirming the contribution of elevated SMPDL3b expression to podocyte injury during DN. These findings indicate that regulation of SMPDL3b expression in different ways may be a therapeutic strategy for podocytopathies under various pathological conditions.

**Fig. 3.**
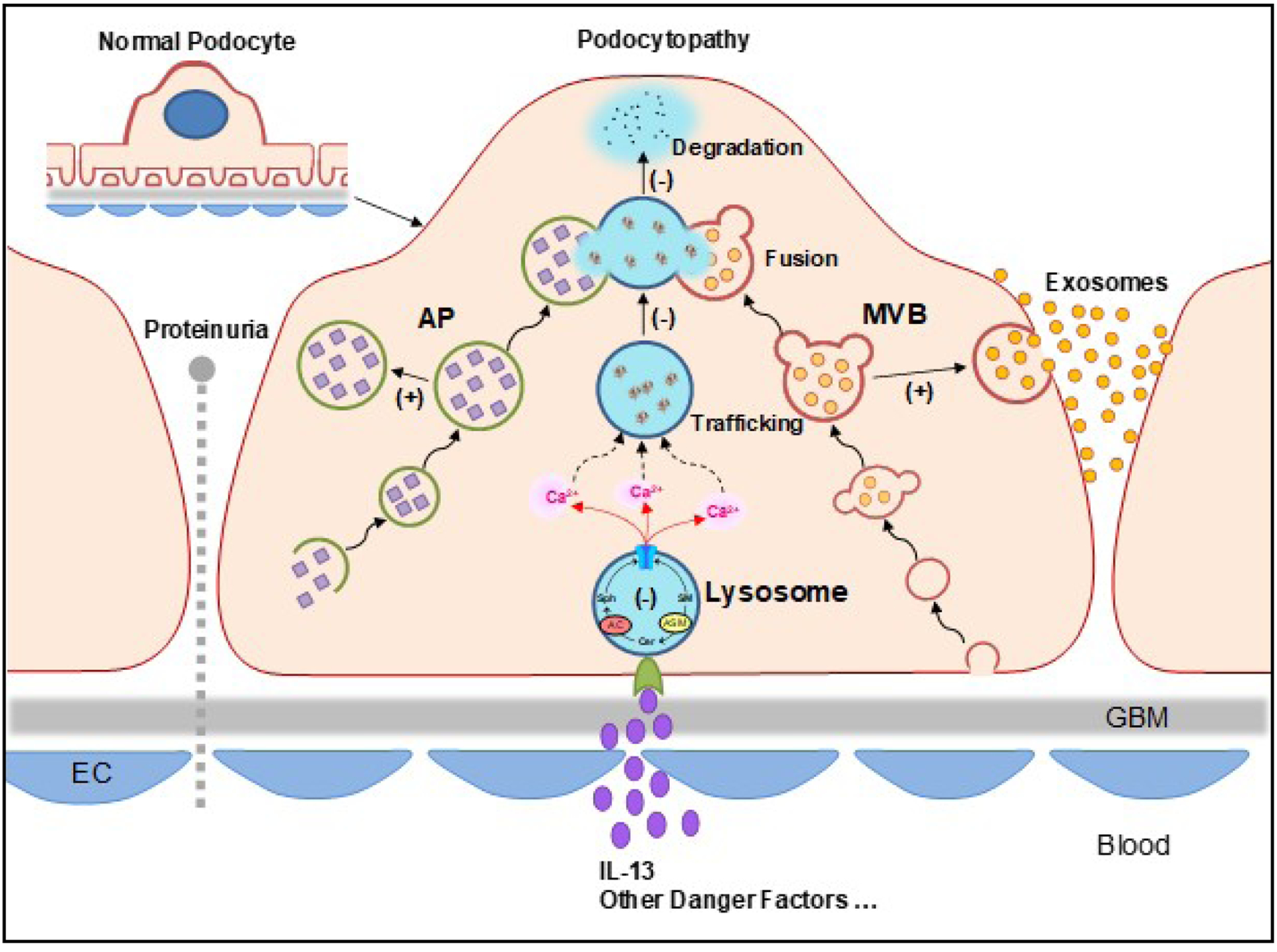
Regulation of lysosome function by sphingolipids and modulating enzymes in podocytes. Lysosome trafficking and fusion to autophagosome and MVB are dependent on the TRPML1 channel-mediated Ca^2+^ release in podocytes. Lysosomal ASM converts SM into CER and AC converts CER to Sph. These sphingolipids, SM, Cer, and Sph had different effects on TRPML1 channel activity in podocytes, with inhibition by SM, no effect from Cer, but enhancement by Sph. SM, sphingomyelin; Cer; ceramide; Sph, sphingosine.

Although reduction of SMPDL3b expression has been found to be associated with decreased ASM activity in podocytes during FSGS, the interaction between SMPDL3b and ASM remains unclear. A recent study has demonstrated that both inhibition and enhancement of SMPDL3b expression increased the level of ASM in podocytes [135]. Interestingly, it was found that ceramide-1-phosphate (C1P) was increased by gene silencing of SMPDL3b but decreased by overexpression of SMPDL3b in podocytes [135]. The binding of SMPDL3b to ceramide kinase blocks the access of ceramide kinase to ceramide and thereby inhibits the conversion of ceramide to C1P [135]. In another study, SMPDL3b dephosphorylated C1P to ceramide [136]. Since C1P is an important signaling molecule, the regulation of C1P metabolism may be a potential molecular mechanism mediating the action of SMPDL3b on the actin cytoskeleton in podocytes. In Fig. 3, the regulation of slit diaphragm and actin cytoskeleton by sphingolipids and associated modulating enzymes in podocytes is illustrated, and these regulatory mechanisms may play fundamental roles in the pathogenesis of NS.

### S1P Lyase

The sphingolipid metabolite, S1P, regulates cell migration, differentiation, and survival by the activation of S1P receptors, a family of five G protein-coupled receptors termed S1P_1_-S1P_5_ [137–140]. The activation of S1P receptors by S1P is involved in various signaling pathways including mitogen-activated protein kinase, c-Jun N-terminal kinase, extracellular signal-regulated kinase, phosphoinositide 3 kinase, phospholipase C, and phospholipase D [141–145]. The expression of S1P_1_, S1P_2_, S1P_3_, and S1P_4_, but not S1P_5_, has been confirmed in podocytes [146]. Therapeutically, it has been reported that renal ischemia-reperfusion injury can be inhibited by the enhancement of S1P synthesis via SK, treatment with S1P_1_ receptor agonist, and delivery of SK gene [147–150]. In addition, the graft rejection in preclinical models of kidney transplantation has been found to be prevented by S1P_1_ receptor agonists [151, 152]. On the contrary, there is evidence that increased S1P production may result in the enhancement of podocyte migratory phenotype after knockout of SMPDL3b gene [134].

For many years, it remains unknown whether S1P lyase is involved in the regulation of podocyte function or in the pathogenesis of podocytopathy and NS. The evidence from recent studies enhanced our understanding regarding the physiological and pathophysiological roles of S1P lyase in podocytes. In tamoxifen-inducible S1P lyase–deficient mice, it was found that even partial deficiency of S1P lyase led to podocyte foot process effacement and proteinuria [41]. A recent clinical study further confirmed that functional deficiency of S1P lyase caused steroid-resistant NS, where 9 different recessive mutations in SGPL1, the gene encoding S1P lyase, were identified in 7 families with steroid-resistant NS [39]. Correspondingly, other clinical studies also confirmed that loss-of-function mutations in S1P lyase caused podocyte injury and steroid-resistant NS [40, 153, 154]. Based on these reports, it seems that the role of S1P in podocyte injury is complex and the precise mechanism by which S1P accumulation induces NS remains unclear. Given that ceramide level was also found increased in S1P lyase–deficient mice, it is possible that S1P accumulation leads to elevation of ceramide, sphingomyelin, or other upstream ceramide substrates, which may contribute to podocyte injury and NS.

## Potential Therapeutic Strategies for NS by Targeting Sphingolipid Metabolism

The goal of therapy for NS is the recovery of podocyte function and reduction of proteinuria, leading to improvement in renal function and resolution of edema, hypoalbuminemia and hyperlipidemia [155]. Angiotensin converting enzyme inhibitors are used to decrease proteinuria in patients but do not affect podocyte structure or function. In diabetic patients, control of blood sugars slows the progression of DN. In addition to symptomatic treatment, therapy targeting podocytes is more specific and effective against certain types of NS. For example, corticosteroid is a canonical therapy for steroid-sensitive NS. However, the relapse of NS after steroid therapy and adverse effects of steroid treatment are disadvantages which remain to be solved. Moreover, steroid resistance has been found in some patients with NS. Other immunosuppressing drugs have been utilized with varying success. Many studies attempted to identify new therapeutic targets and develop new strategies for the treatment of NS. In this regard, targeting sphingolipids may be a potential strategy for more effective treatment of NS, in particular, steroid-resistant NS.

The role of B cells in the pathogenesis of MCD and FSGS has gained attention due to the successful use of B cell depleting agents. As a chimeric monoclonal antibody against the protein CD20, rituximab exerts its B cell-depleting effect via binding to CD20 on the surface of B cells. Because complete remission of NS unexpectedly occurs in patients with post-transplant recurrent FSGS treated with rituximab, this monoclonal antibody has been a promising candidate drug for treating recurrent or refractory FSGS [156–159]. A recent study revealed that human glomeruli including podocytes expressed neither CD20 mRNA nor protein and that rituximab restored actin cytoskeleton through B cell-independent mechanisms. Rituximab may act through 3 different therapeutic mechanisms against NS. First, rituximab induces depletion of B-cells and inhibits antigen presentation and activation of antigen by T cells through interaction with the T-cell receptor. Consequently, rituximab results in decreased production of cytokines that might increase glomerular permeability. Second, rituximab-induced enhancement of cytotoxic T-lymphocyte protein 4 production by T regulatory cells may inhibit CD80 activation in podocytes, leading to remission of proteinuria. In the third mechanism, SMPDL3b as an unexpected target of rituximab plays pivotal role in the treatment of FSGS. After the discovery that rituximab binds to SMPDL3b in podocytes [160], a recent study revealed that human glomeruli including podocytes expressed neither CD20 mRNA nor protein and that rituximab restored podocyte actin cytoskeleton through B cell-independent mechanisms [132]. Moreover, reduction of SMPDL3b expression and ASM activity were found to contribute to actin stress fiber formation and actin cytoskeletal disorganization in podocytes after exposure to the sera of patients with recurrent FSGS. Therapy with rituximab reversed these pathological changes [132]. In another study, radiation-induced downregulation of SMPDL3b in podocytes led to reduction of S1P production, cytosolic translocation of ezrin, and actin cytoskeleton remodeling in these cells, which were prevented by rituximab [161]. These findings indicate that podocyte SMPDL3b may be the therapeutic target of rituximab against NS in which pathogenesis is within podocytes. However, many questions remain to be answered for further development of this therapy. For example, the substrates and products of SMPDL3b still remain unknown, although high resolution crystal structure of murine SMPDL3b reveals a substrate binding site strikingly different from its paralog, ASM [162]. In studies on human podocytes, SMPDL3b was found to modulate the activity of ASM and regulates the generation of ceramide, but the molecular mechanism remains unclear [132]. More recently, it has been demonstrated that SMPDL3b dephosphorylates C1P to ceramide and inhibits the function of ceramide kinase [135, 136], but it remains unknown whether these functions are involved in the action of SMPDL3b on podocyte actin cytoskeleton. On the other hand, the limitation of rituximab should not be ignored. Although rituximab has been reported in some studies to have high success potential to induce the remission of NS, summarized data showed that remission of NS only occurred in 64.7% of MCD patients and 31.3% of FSGS patients [163]. It is clear that more studies are needed to develop medications to better or more effectively block SMPSL3b downregulation or inhibition during FSGS compared to rituximab.

As discussed above, AC has recently been reported to be crucial in the regulation of podocyte functional and structural integrity, and its deficiency leads to podocytopathy and NS. Therefore, targeting AC may be a potential therapeutic strategy for treatment of NS, especially steroid-resistant NS. In 2014, studies on Sprague-Dawley rats have shown that soy protein decreases kidney damages in rats with NS. Addition of genistein, an AC activator, to soy protein caused improvements in antioxidant status of kidney tissue, which was associated with inhibition of cell proliferation [164]. Further studies demonstrated that genistein significantly decreased low-density lipoprotein cholesterol and interleukin-6 in rats with NS [165]. These findings indicate a potential role of genistein in therapy of NS by improving the systemic environment for glomeruli. However, there is a lack of evidence about the therapeutic effects of genistein on podocyte injury during NS, despite that podocytopathy and NS occur in mice with podocyte-specific Asah1 gene deletion. In addition to AC, molecular targets of genistein include caspases, B-cell lymphoma 2, Bcl-2-associated X protein, phosphoinositide 3-kinase/Akt, extracellular signal-regulated kinase 1/2, mitogen-activated protein kinase, and Wingless and integration 1/β-catenin signaling pathway [166]. Therefore, new activators of AC with high efficacy and selectivity are required for clinical usage. For treatment of NS due to genetic mutations of AC, enzyme replacement therapy is an alternative approach. The human recombinant AC overexpressed by Chinese hamster ovary cells were used to treat fibroblasts from a Farber disease patient, leading to significant reduction of ceramide. The administration of human recombinant AC to mice with Farber disease confirmed the enzymatic activity *in vivo*. The ceramide level was maintained in a normal range for at least 7 days after enzyme administration [167]. These results suggest that enzyme replacement therapy should be further developed for therapy of NS due to genetic mutation of AC.

It is expected that more therapeutic strategies will be forthcoming, which include the use of ASM inhibitor, AC inducer, SMPDL3b activator, and α-GLA inducer. Also, development of enzyme replacement therapy may be important for treatment of idiopathic NS due to genetic mutations. These potential therapeutics target different sphingolipids and modulating enzymes in podocytes, which may be selected for the use in the prevention or treatment of NS.

## Concluding Remarks

In this review, we briefly summarize the current evidence about molecular mechanisms by which sphingolipid metabolism affects podocyte function and integrity, including the involvement of ganglioside, Gb3, ceramide, AC, ASM, SMPDL3b, and S1P lyase. All these studies have provided innovative insights into pathogenesis of NS and potential to prevent or treat NS by targeting abnormal sphingolipid metabolism. The imbalance of sphingolipid metabolism in podocytes may be induced by genetic mutations or systemic disorders. Therefore, sphingolipids and modulating enzymes have been implicated in the development of a variety of NS due to their induction of podocyte dysfunction and injury. Further mechanistic investigations are of the utmost importance to understand how various sphingolipid signaling pathways interact to regulate structural and functional integrity of podocytes, which may promote the development of more effective therapies for the prevention or treatment of NS.

## Figures and Tables

**Table 1.** Pathogenesis-based classification of podocytopathies.

Type of Podocytopathy	Cause and Pathogenesis	Pathology	Clinical Manifestation	Treatment
Permeability factor induced	Unknown circulating factor causing podocyte injury	MCD, FSGS, podocyte foot process effacement	Nephrotic syndrome	Immunosuppressive therapy
Toxic	Direct toxicity, i.e. infectious, drug induced, related to APOL1	MCD, FSGS, can include tubuloreticular inclusions	Variable	Removal of toxin, supportive therapy
Genetic	Mutation leading to abnormality at the podocyte	MCD, FSGS, mesangioproliferative GN	Steroid resistant NS	Supportive therapy, renin-angiotensin blockers
Hyperfiltration mediated	Adaptive changes due to excessive nephron workload—obesity, hypoxia, decreased renal mass	FSGS, segmental foot process effacement	Slowly progressive chronic kidney disease, proteinuria without hypoalbuminemia	Supportive therapy, renin-angiotensin blockers

MCD, minimal change disease; FSGS, focal segmental glomerulosclerosis; GN, glomerulonephritis; NS, nephrotic syndrome. This table is adapted from Ahn W, Bomback AS: Approach to Diagnosis and Management of Primary Glomerular Diseases Due to Podocytopathies in Adults: Core Curriculum 2020. Am J Kidney Dis 2020;75:955–964
